# Pediatric rhabdomyolysis: a systematic review and meta-analysis of etiologies, management, and outcomes

**DOI:** 10.1186/s12887-025-06081-x

**Published:** 2025-10-27

**Authors:** Hany A. Zaki, Hussam Elmelliti, Waseem Ahmad Malik, Eman E. Shaban, Amira Shaban, Ahmed Shaban

**Affiliations:** 1 College of Medicine, Qatar University, Doha, Qatar; 2 Emergency Medicine Department, Hamad Medical Corporation, Doha, Qatar; 3 Cardiology Department, Al Jufairi Diagnosis and Treatment, MOH, Doha, Qatar; 4 Internal Medicine Department, Mansoura University Hospital, El Mansoura, Egypt

**Keywords:** Rhabdomyolysis, Pediatric patients, Chronic kidney disease, Acute kidney injury

## Abstract

**Background:**

Rhabdomyolysis is a potentially fatal disorder that occurs due to various causes. Therefore, the aim of this meta-analysis was to identify the underlying causes, evaluate the treatment options, and determine the mortality and kidney outcomes of children with rhabdomyolysis.

**Methods:**

We comprehensively searched PubMed, Cochrane Library, Web of Science, EMBASE, and Google Scholar databases for records written in English and published until April 2025. According to PICO criteria, we included studies that enrolled pediatric patients with rhabdomyolysis and reported etiologies, treatments, mortality, and/or kidney outcomes. Subsequently, quality appraisal was conducted with Newcastle Ottawa Scale and statistical analyses were performed using comprehensive meta-analysis (CMA) software.

**Results:**

Fifteen studies enrolling 10,514 pediatric patients with rhabdomyolysis were reviewed and analyzed. The one-arm meta-analysis revealed that infections were the most predominant etiology of rhabdomyolysis (40.6%; 95% CI: 33.5 to 48.2), followed by trauma (19%; 95% CI: 15.5 to 23.0) and exercise (14.7%; 95% CI: 6.5 to 30.2). Other etiologies accounted for less than 10% of rhabdomyolysis cases i.e., burns (2.9%; 95% CI: 1.2 to 7.2), connective tissue disorder (2.7%; 95% CI: 0.7 to 9.6), drugs (5.8%; 95% CI: 3.1 to 10.6), metabolic abnormalities (4.4%; 95% CI: 3.0 to 6.6), multiorgan failure (4.1%; 95% CI: 1.0 to 15.5), muscular dystrophy (2.6%; 95% CI: 0.6 to 11.4), seizure (7.2%; 95% CI: 3.9 to 12.9), and sepsis (9.9%; 95% CI: 1.1 to 52.8). The pooled results also showed that the incidences of acute kidney injury (AKI) and chronic kidney disease (CKD) were 21.3% (95% CI: 14.5 to 30.3; I^2^ = 96%) and 1.1% (95% CI: 0.7 to 2; I^2^ = 0%), respectively. The pooled mortality rate of children with rhabdomyolysis was 4.5% (95% CI: 1.7 to 11.8; I^2^ = 94.7%).

**Conclusions:**

Infections are the leading causes of rhabdomyolysis in children. Moreover, AKI is a common complication of rhabdomyolysis in children. However, the prognosis of children with rhabdomyolysis is good and few patients progress to CKD.

**Systematic review protocol registration:**

PROSPERO: CRD420251035968.

**Supplementary Information:**

The online version contains supplementary material available at 10.1186/s12887-025-06081-x.

## Introduction

Rhabdomyolysis is a systemic metabolic illness, with roughly 26,000 events recorded per year in the United States alone [1]. This syndrome is typically marked by skeletal muscle necrosis which leads to leaking of muscle cell components, such as myoglobin, into the bloodstream. The disorder may appear as an asymptomatic illness with high creatine kinase (CK) values or become a serious health issue with significant elevation in CK, electrolyte deficiencies, acute kidney injury (AKI), and disseminated intravascular coagulation [1, 2]. Clinically, rhabdomyolysis is characterized by a triad of symptoms, comprising myalgia, weakness, and myoglobinuria, which appears as the generally recognized tea-colored urine. However, this stringent description of symptoms may be deceiving since the trio of symptoms is only found in < 10% of patients, and > 50% of patients have no complaints of muscular pain or weakness, despite presenting with stained urine [3–5]. Therefore, elevated CK level is an exceptionally sensitive laboratory test for detecting muscular injury that has the potential to produce rhabdomyolysis, providing there is no concomitant heart or brain damage.

In adults, the available literature has shown that the most common causes of rhabdomyolysis are drug or alcohol abuse, medicinal drug use, trauma, immobility, and neuroleptic malignant syndrome [6–8]. However, the data in pediatric patients is skewed towards different causes, with viral myositis, trauma, connective tissue disorders, exercise, and drug overdose being reported as the leading causes of rhabdomyolysis in this population [8]. Moreover, the treatment strategies for pediatric rhabdomyolysis remain an area of ongoing clinical debate. Therefore, the present systematic review and meta-analysis was designed to identify and categorize etiologies of pediatric rhabdomyolysis, evaluate the different treatment strategies for rhabdomyolysis, and assess the outcomes of children with rhabdomyolysis.

## Methods

### Protocol and registration

This systematic review was conducted according to the guidelines of PRISMA 2020 (Preferred Reporting Items for Systematic Reviews and Meta-Analyses). The completed PRISMA 2020 checklist is provided in Supplementary Material 2. The protocol for this systematic review was pre-registered with the International Prospective Register of Systematic Reviews (PROSPERO; registration number [CRD420251035968]). The registration includes the review objectives, inclusion/exclusion criteria, data sources, and planned analysis methods. The full protocol can be accessed at:

https://www.crd.york.ac.uk/PROSPERO/view/CRD420251035968.

## Literature searches and information sources

We comprehensively searched PubMed, Cochrane Library, Web of Science, EMBASE, and Google Scholar databases for records published until April 2025. This online database search was limited to English-published records and involved combining several keywords with the Boolean Expressions “AND” and “OR” to form a comprehensive search strategy. The keywords used included pediatric, rhabdomyolysis, etiology, acute kidney injury, mortality, and management. A manual search, which consisted of hand-searching the reference lists of previously published literature reviews and other relevant documents, was also conducted to supplement the online search. During the search, we excluded grey literature, such as theses and dissertations, as we only needed to conduct analysis on published data. The complete search criteria across the mentioned databases are displayed in Appendix A (Supplementary Material 1).

## Eligibility criteria

Studies were deemed eligible for inclusion if they satisfied the following PICO criteria: Patients (P): Pediatric patients diagnosed with rhabdomyolysis. Interventions (I): Different treatment options for rhabdomyolysis, such as bicarbonate, mannitol, hydration, and hemodialysis. Comparison (C): no intervention, controls, or patients without rhabdomyolysis. Outcomes (C): causes of rhabdomyolysis, incidence of AKI, and mortality rates. The following PECO criteria was also used in identifying studies to be included in the current meta-analysis: Population (P): Children with rhabdomyolysis. Exposure (E): Specific etiologies for rhabdomyolysis, such as infections, drugs, trauma, metabolic disorders, etc. Comparison (C): Other etiologies Outcomes (C): Complications, AKI, CKD, and mortality rates.

The exclusion criteria were as follows:

1. Studies involving adult patients.
2. Studies designed as case reports, editorials, literature reviews, conference abstracts, letters to the editor, or animal studies.
3. Studies with less than 10 participants. This particular criterion was used to improve the statistical power of our meta-analysis, since studies with very small sample sizes are likely to overestimate or underestimate the overall effect size.
4. Studies reporting only the diagnostic criteria of rhabdomyolysis.

## Data extraction

Two authors independently screened through the full-text records and extracted all the necessary data for review and analysis on a standard Excel spreadsheet. The data extracted from each study included the name of the primary author, date of publication, the study’s geographical region, total number of patients with rhabdomyolysis, the mean/median age, gender distribution, definition of rhabdomyolysis, treatment strategies, etiologies of rhabdomyolysis, and outcomes of patients with rhabdomyolysis (i.e., mortality rate and incidence of AKI and chronic kidney disease (CKD)). All the discrepancies in the extracted data were resolved through debates between the reviewers or by soliciting the input of a third reviewer.

## Quality appraisal

All included studies were observational; therefore, we investigated the methodological quality of each study using the Newcastle Ottawa Scale (NOS) [9], which is suitable for such studies. With this tool, the studies were assigned scores based on three domains, namely selection, comparability, and outcome. Thereafter, each study was graded as having poor, fair, or high methodological quality according to the total NOS scores (NOS scores between 0 and 3, 4–6, and 7–9, respectively).

### Data synthesis

The Comprehensive Meta-Analysis (CMA version 3.0) software was used for all statistical analyses in the current study. Data regarding the etiologies of rhabdomyolysis, mortality and AKI were pooled in a one-arm meta-analysis and computed using the untransformed (raw) proportion. The DerSimonian-Laird random effects model was fitted to all pooled outcomes as it accounts for heterogeneity and provides conservative estimates. In addition, interstudy heterogeneity was quantified using I^2^ statistics, of which index values of more than 50% indicated significant heterogeneity [10, 11]. All meta-analysis results were computed with their corresponding 95% confidence intervals (CIs) and visually presented using forest plots. Whenever possible subgroup analyses were conducted according to the diagnostic criteria for rhabdomyolysis. A leave-one-out sensitivity analysis was also conducted to investigate the sources of heterogeneity.

## Results

### Study selection

**Fig. 1:**
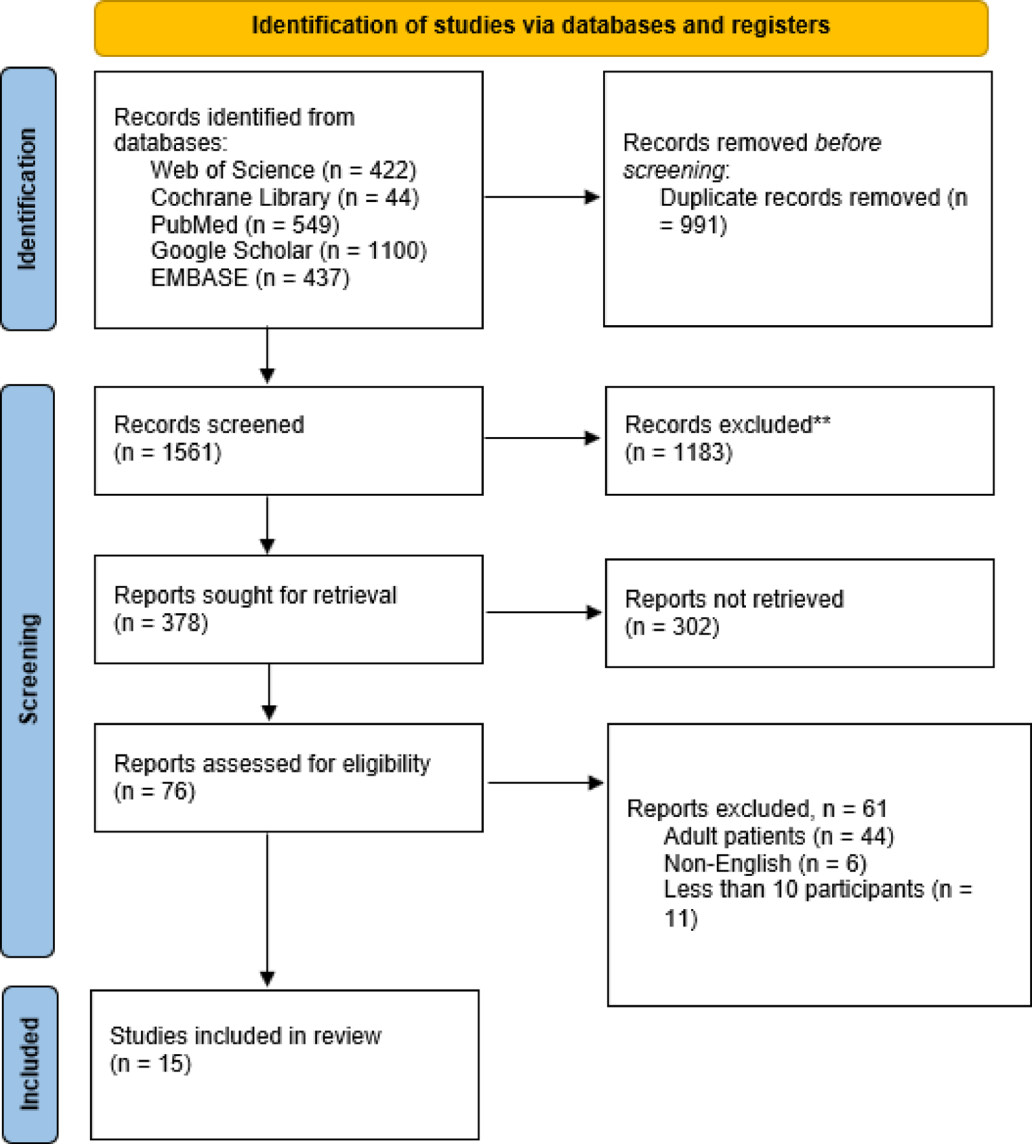
PRISMA flow chart for study selection

A total of 2552 potential studies were identified across the five databases mentioned earlier. 991 of these articles were excluded after they were confirmed to be exact or close duplicates. Another 1183 articles whose titles and abstracts were irrelevant to our study objective were excluded. We also did not retrieve 302 articles designed as case reports, narrative reviews, abstracts without full texts, or letters to the editor. Finally, 15 unique observational studies satisfied the full-text screening and were included in the analysis. The remaining 61 articles were excluded due to the following reasons: 6 were non-English articles, 11 had less than 10 participants, and 44 involved adult patients (Fig. 1).

### Characteristics of included studies

Fifteen studies involving 10,514 children with rhabdomyolysis were included [12–26]. All the studies were retrospective in nature and conducted in single centers. These studies were published between 2000 and 2024. In addition, various definitions were used for rhabdomyolysis. These definitions were based on serum CK levels, serum myoglobin levels, and urine myoglobin level (Table 1).

**Table 1:** Summary of study characteristics

Author ID	Study design	Country	Participant characteristics			Etiologies (No. of patients)	Treatment strategies (No. of patients)	Definition of rhabdomyolysis	Outcomes
			No. of patients	M/F	Mean/ Median Age				
Chen et al. 2013 [12]	Retrospective study	Taiwan	37	26/11	10.2 years	Trauma (6) Exercise (6) Infection (22) Metabolic and electrolyte (2) Body temperature change (1)	Hydration (37)	Serum CK > 1000 IU/L without cardiac etiology or genetic muscular dystrophy.	AKI: 3/37
Park et al. 2018 [13]	Retrospective study	Korea	45	35/10	116 months	Infection (26) Physical exertion (9) Prolonged seizure (1) Metabolic abnormalities (3) Medication (2) Multiorgan failure (4)	Hydration (37) Hydration plus alkalization (2) Dialysis (2)	Serum myoglobin ≥ 80 ng/mL without acute myocardial injury, cardiac arrest and brain damage	AKI: 6/45
Yao et al. 2020 [14]	Retrospective study	China	55	37/18	11 years	Infection (9) Exercise (13) Immunology (9) Genetic (8) Metabolism (2) Poison/drug (4) Trauma (4)	Hydration, alkalization, and/or RRT (6) RRT (12)	Serum CK levels > 1000 IU/L	Mortality: 6/55 AKI: 27/55
Agharokh et al. 2022 [15]	Retrospective study	United States	8599	6911/1688	NR	NR	RRT (35)	NR	AKI: 733/8599 Mortality: 3/8599
Harmer et al. 2023 [16]	Retrospective study	United Kingdom	232	157/75	8.4 years	Infection (64) Trauma (31) Seizures (23) Immune-mediated (19) Toxin/Drugs (18) Metabolic disturbances (12) Exercise (6)	Hydration (77) Alkalinization (3) RRT (18)	Serum CK levels > 1000 IU/L	Mortality: 22/232 AKI: 74/232
Yoo et al. 2021 [17]	Retrospective study	Korea	880	634/246	8 years	Infection (343) Trauma (186) Prolonged seizures (185)	Volume replacement (634) Alkalization (102) Mannitol (34) Dialysis (31)	Serum CK levels > 1000 IU/L or myoglobin level ≥ 100 ng/mL	AKI: 99/880 Mortality: 28/880
Kuok et al. 2021 [18]	Retrospective study	China	54	45/9	7.8 years	Infection (39) Physical exertion (5) Seizures (3) Irritability/dystonia (2)	Hydration (18) Sodium bicarbonate (5)	Serum CK levels > 1000 IU/L	AKI: 10/54 Mortality: 0/54
Mannix et al. 2006 [19]	Retrospective study	United States	191	128/63	11 years	Viral infection (73) Trauma (49) Connective tissue disease (10) Exercise (8) Drug overdose (8) Metabolic (7) Seizure	NR	Serum CK levels > 1000 IU/L without myocardial infarction	AKI: 9/191
Gelbart et al. 2018 [20]	Retrospective study	Australia	182	117/65	100 months	Sepsis (47) Septic shock (35) Cardiac arrest (40) Trauma (39) Seizures (10) Malignancy (6) Burn (8) Electrocution (2) Electrolyte imbalance (3) Drug (4) Metabolic disturbance (6) Envenomation (3)	RRT (29)	Serum CK levels > 1000 IU/L	Mortality: 36/182 AKI: 36/182
Watanabe, 2001 [21]	Retrospective study	Japan	18	14/4		Infection (11) Seizure (3) Metabolic (1)	NR	Serum myoglobin > 300 ng/mL and urinary myoglobin > 10 ng/mL	Mortality: 6/18 AKI: 9/18
Watemberg et al. 2000 [22]	Retrospective study	Israel	19	8/11	11 years	Trauma (5) Viral myositis (2) Hyperosmolarity (2) Malignant hyperthermia (2) Exertion (1) Polymyositis (1) Metabolic (1) Hypovolemia (1) Seizure (1) Viral illness (1) Hypernatremia (3)	NR	NR	AKI: 8/19
Zepeda-Orozco et al. 2008 [23]	Retrospective study	United States	28	19/9	11.1 years	Infection (12) Exertion (7) Trauma (3) Diabetic ketoacidosis (1) Seizure (1) Toxin (1)	RRT (7)	Serum CK > 1000 IU/L without cardiac etiology or genetic muscular dystrophy	AKI: 11/28 Mortality: 0/28
Lim et al. 2018 [24]	Retrospective study	Korea	39	28/11	14 years	Infection (12) Exercise (9) Trauma (8) Ischemia (4) Drug (4) Seizure (2)	RRT (5)	Serum CK levels > 1000 IU/L and serum myoglobin level > 150 ng/mL without myocardial infarction or inflammatory myopathies	Mortality: 0/39 AKI: 14/39
Pinto et al. 2024 [25]	Retrospective study	United States	112	89/23	13.5 years	Exertion (67) Viral infections (36)	Sodium bicarbonate (26)	Serum CK levels > 1000 IU/L	AKI: 8/112
Azapağası et al. 2022 [26]	Retrospective study	Turkey	23	12/11	70 months	Intoxication (5) Infection (11) Metabolic (4)	RRT (6)	Serum CK levels > 1000 IU/L	AKI: 11/23 Mortality: 4/23

NR: Not Reported

### Etiologies

**Fig. 2:**
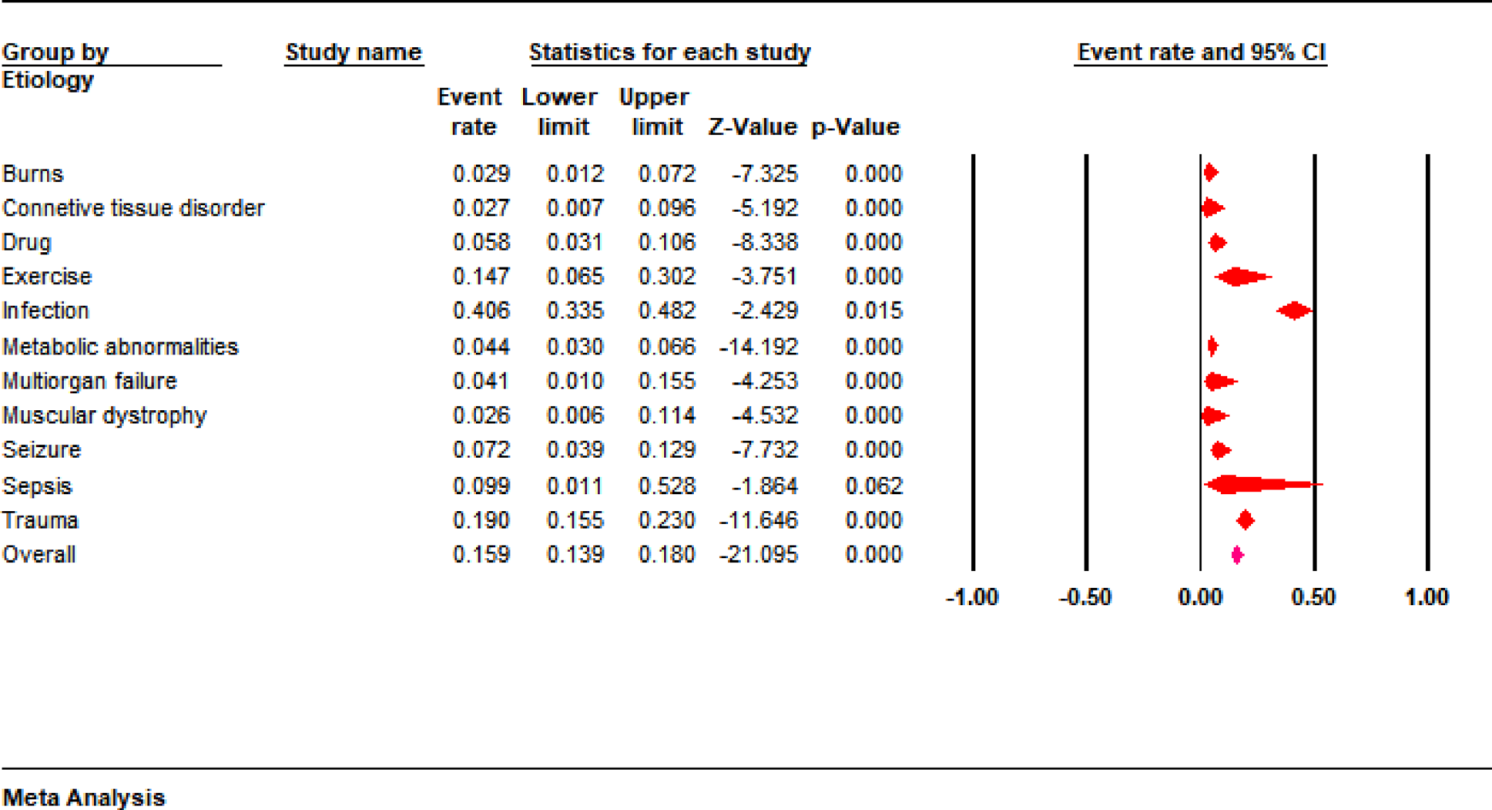
Forest plots showing etiologies of rhabdomyolysis

**Fig. 3:**
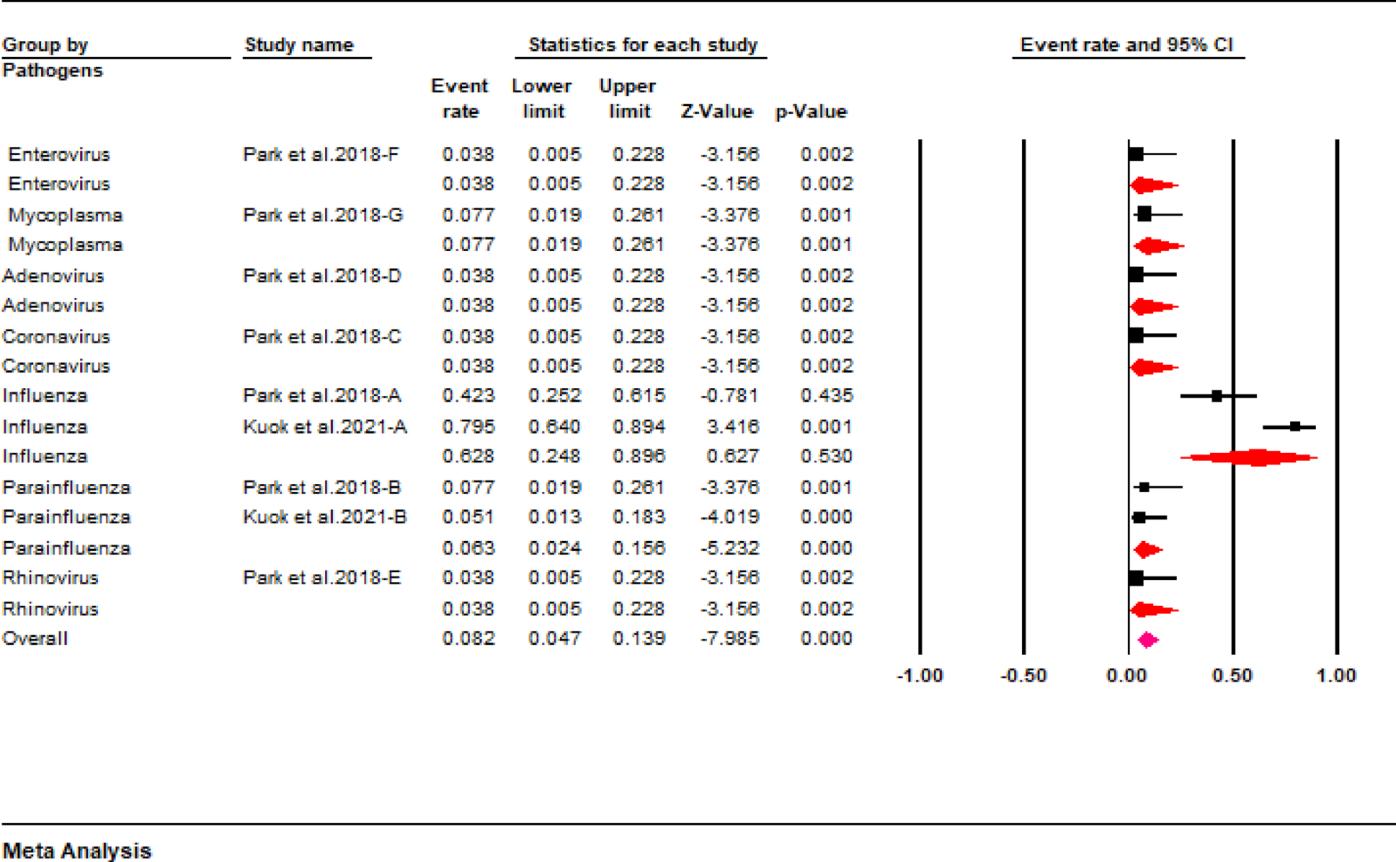
Forest plot showing pathogens causing infection-related rhabdomyolysis

The pooled results showed that the most common cause of rhabdomyolysis in pediatric patients was infection (40.6%; 95% CI: 33.5 to 48.2), followed by trauma (19%; 95% CI: 15.5 to 23) and exercise (14.7%; 95% CI: 6.5 to 30.2). The other etiologies accounted for less than 10% of rhabdomyolysis cases i.e., burns (2.9%), connective tissue disorder (2.7%), drugs (5.8%), metabolic abnormalities (4.4%), multiorgan failure (4.1%), muscular dystrophy (2.6%), seizure (7.2%), and sepsis (9.9%) (Fig. 2). A subgroup analysis according to different pathogens revealed that the influenza virus is a leading cause of infection-related rhabdomyolysis (62.8%; 95% CI: 24.8 to 89.6). Other pathogens involved in the development of infection-related rhabdomyolysis were parainfluenza (6.3%), coronavirus (3.8%), adenovirus (3.8%), rhinovirus (3.8%), enterovirus (3.8%), and mycoplasma (7.7%) (Fig. 3).

### Mortality and kidney outcomes

**Fig. 4:**
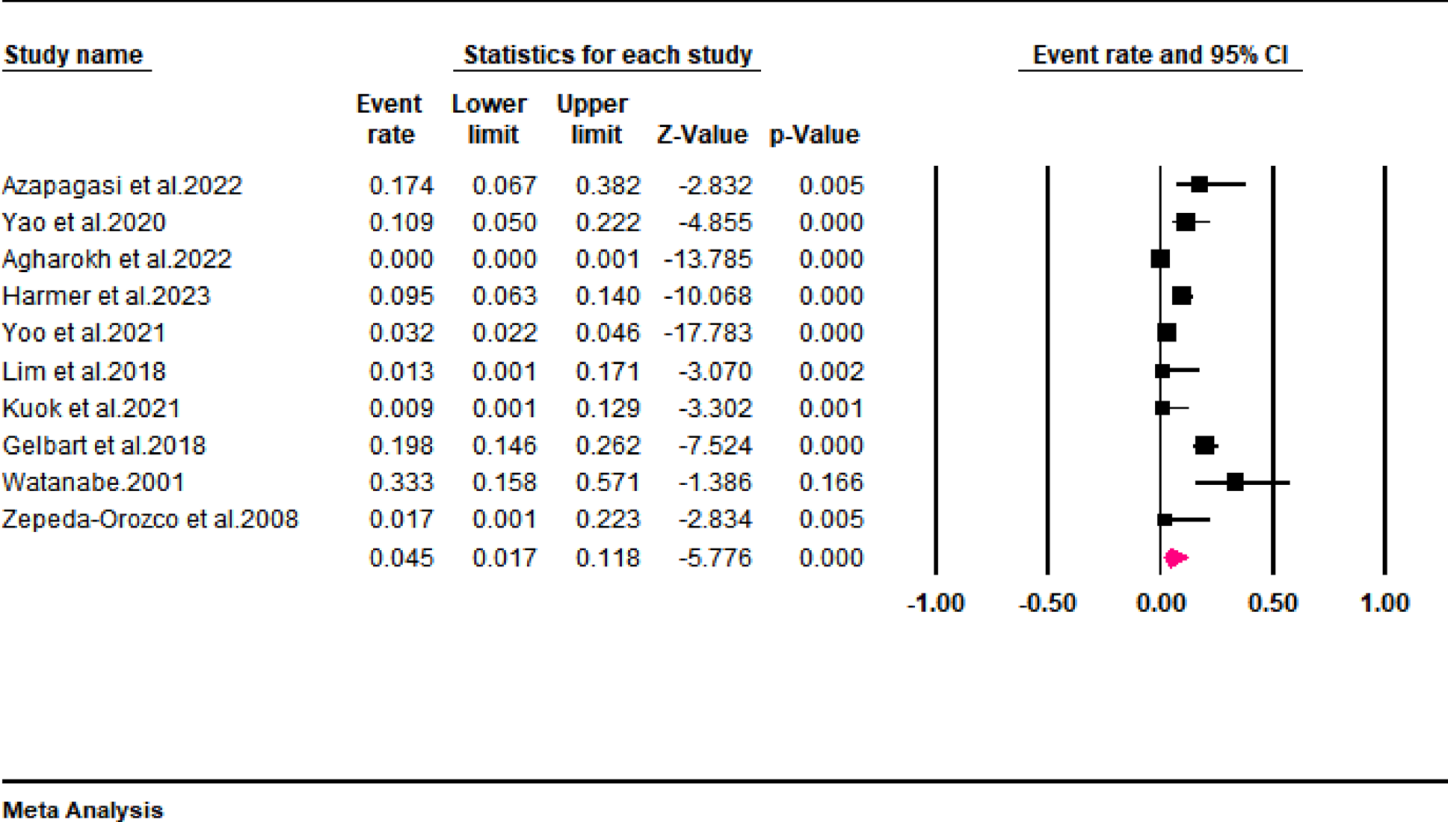
Forest plot showing mortality rate

**Fig. 5:**
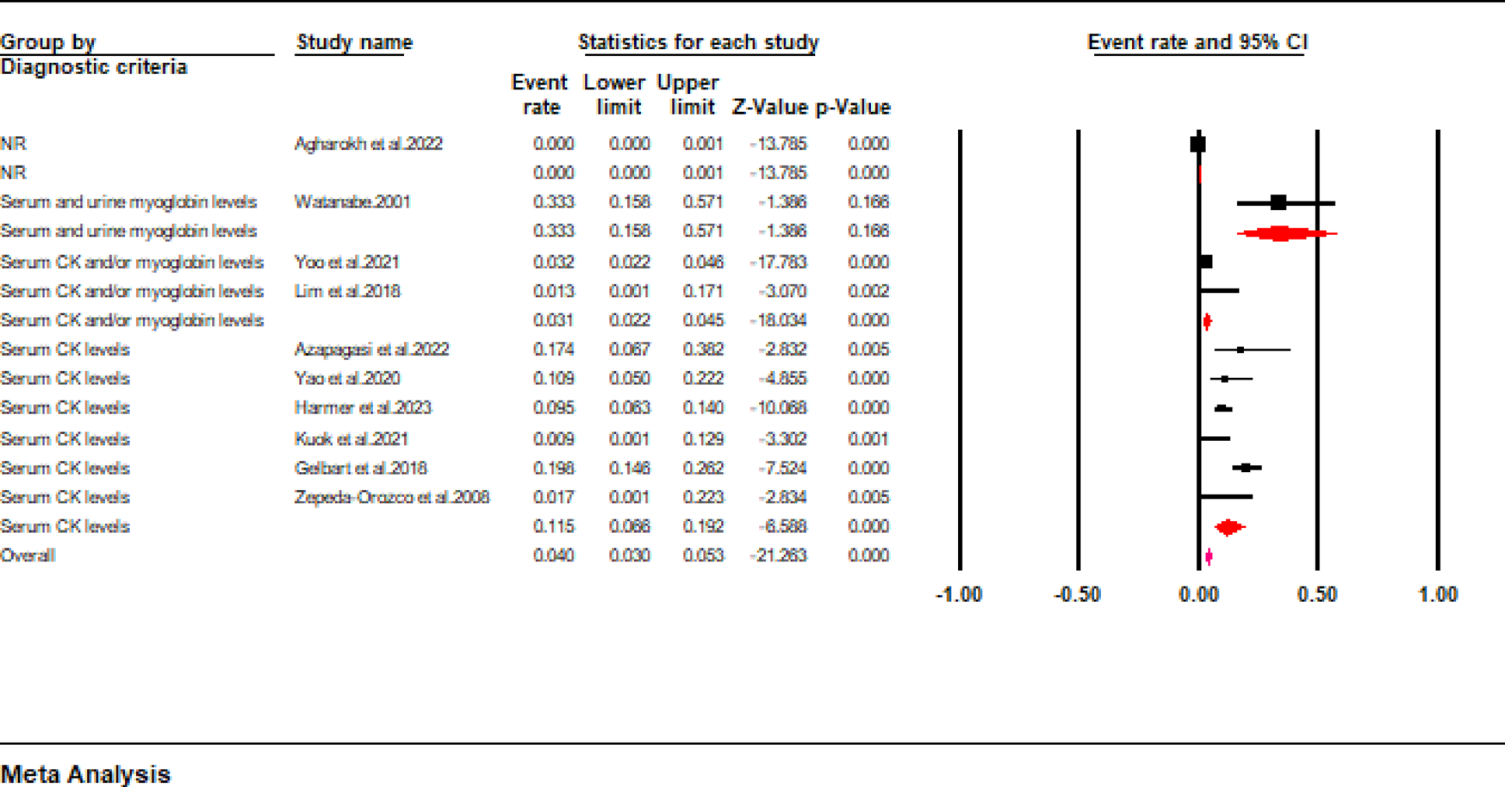
Forest plot showing mortality rate stratified according to the diagnostic criteria

Mortality outcomes were only reported in ten studies included in the current review. Data pooled from these studies revealed that the mortality rate of pediatric patients with rhabdomyolysis was approximately 4.5% (95% CI: 1.7 to 11.8) (Fig. 4). However, a high interstudy heterogeneity was noted in the pooled results (I^2^ = 94.7%). When a subgroup analysis was conducted according to the diagnostic criteria, we found that the pooled mortality rates were 33% (95% CI: 15.8 to 57.1), 3.1% (95% CI: 2.2 to 4.5), and 1.7% (95% CI: 0.1 to 22.3) for studies using serum and urinary levels, serum CK and/or myoglobin levels, and serum CK levels as the criteria for diagnosing rhabdomyolysis (Fig. 5).

**Fig. 6:**
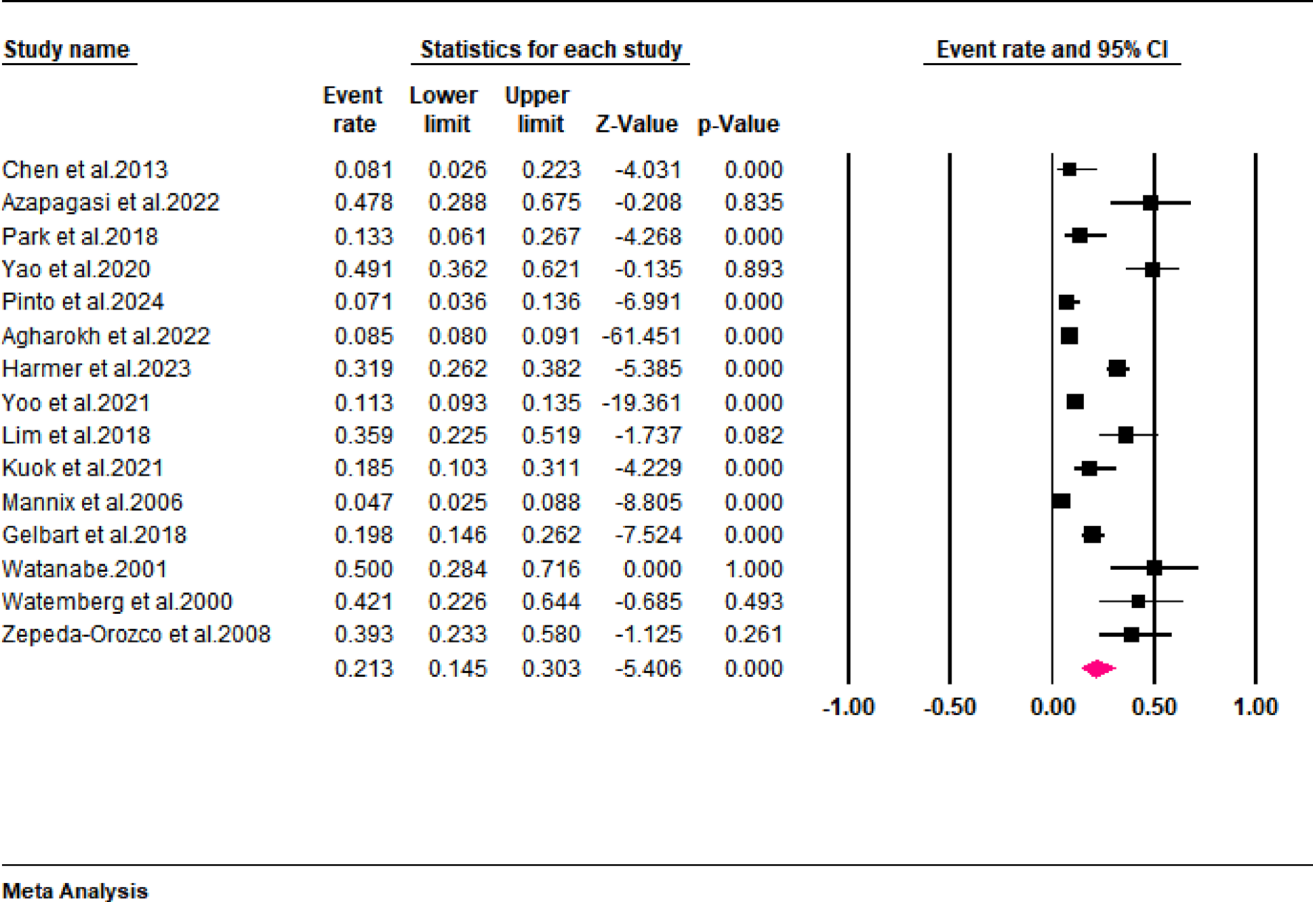
Forest plot showing the incidence of AKI

**Fig. 7:**
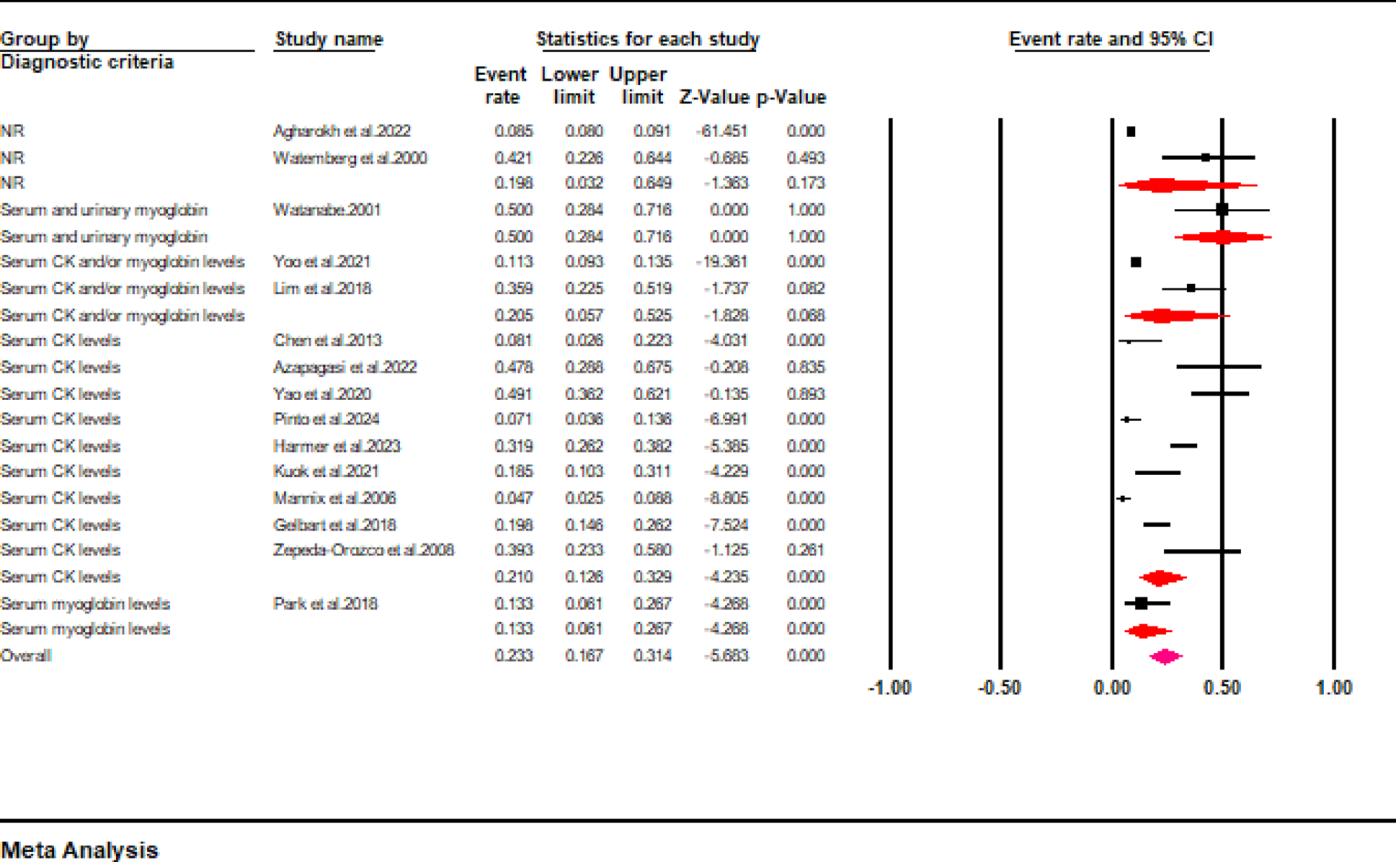
Forest plot showing the incidence of AKI stratified according to diagnostic criteria

In addition, the pooled results revealed that AKI occurred in about 21.3% (95% CI: 14.5 to 30.3) of pediatric patients with rhabdomyolysis (Fig. 6). However, this finding demonstrated a high interstudy heterogeneity (I^2^ = 96%). Further analysis stratified according to the diagnostic criteria revealed that the incidences of AKI were 50% (95% CI: 28.4 to 71.6), 20.5% (95% CI: 5.7 to 52.5), 21% (12.6 to 32.9), and 13.3% (95% CI: 6.1 to 26.7) when rhabdomyolysis was diagnosed based on serum and urinary myoglobin levels, serum CK and/or myoglobin levels, serum CK levels, and serum myoglobin levels, respectively (Fig. 7).

**Fig. 8:**
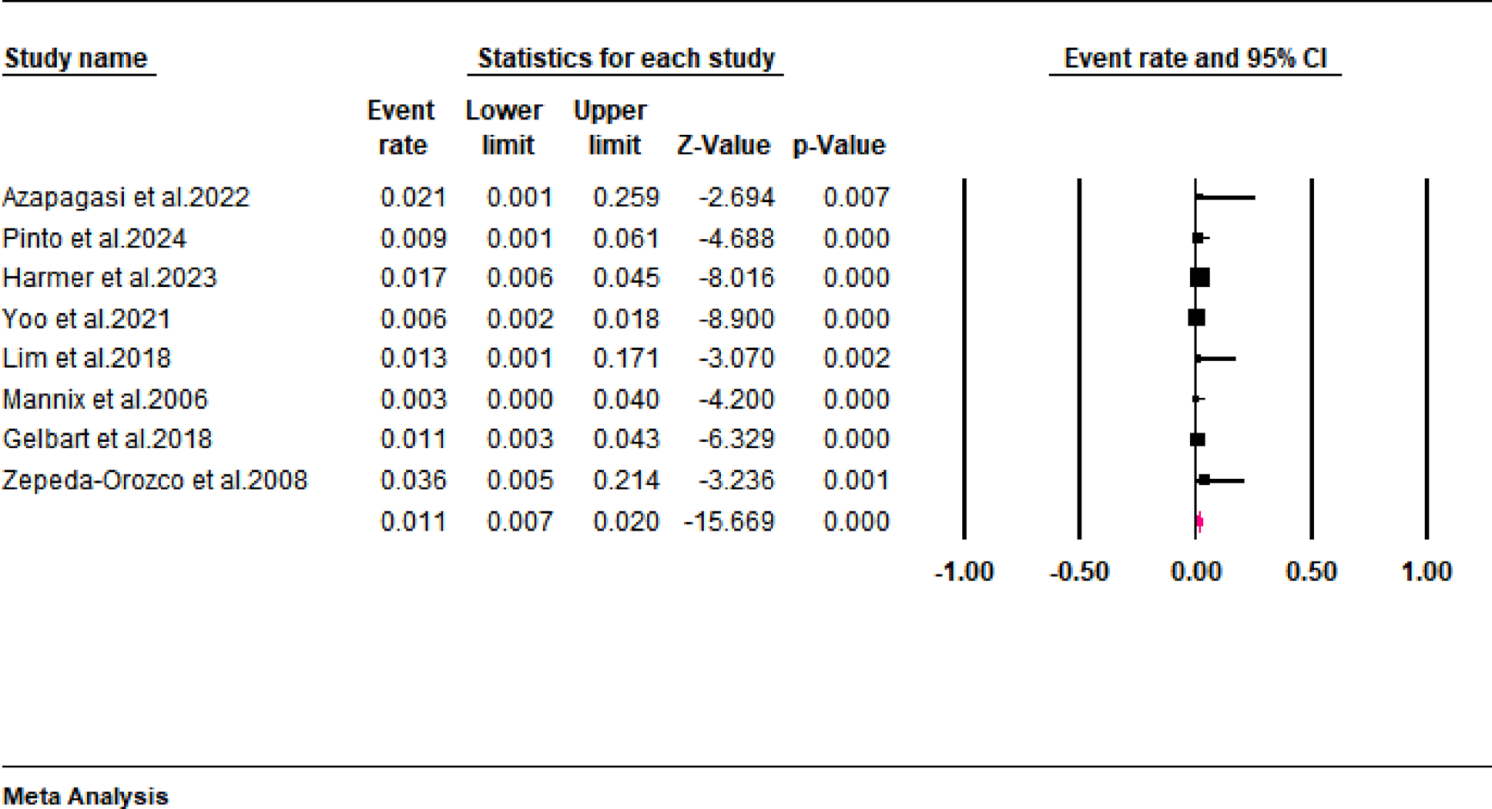
Forest plot showing the incidence of CKD

**Fig. 9:**
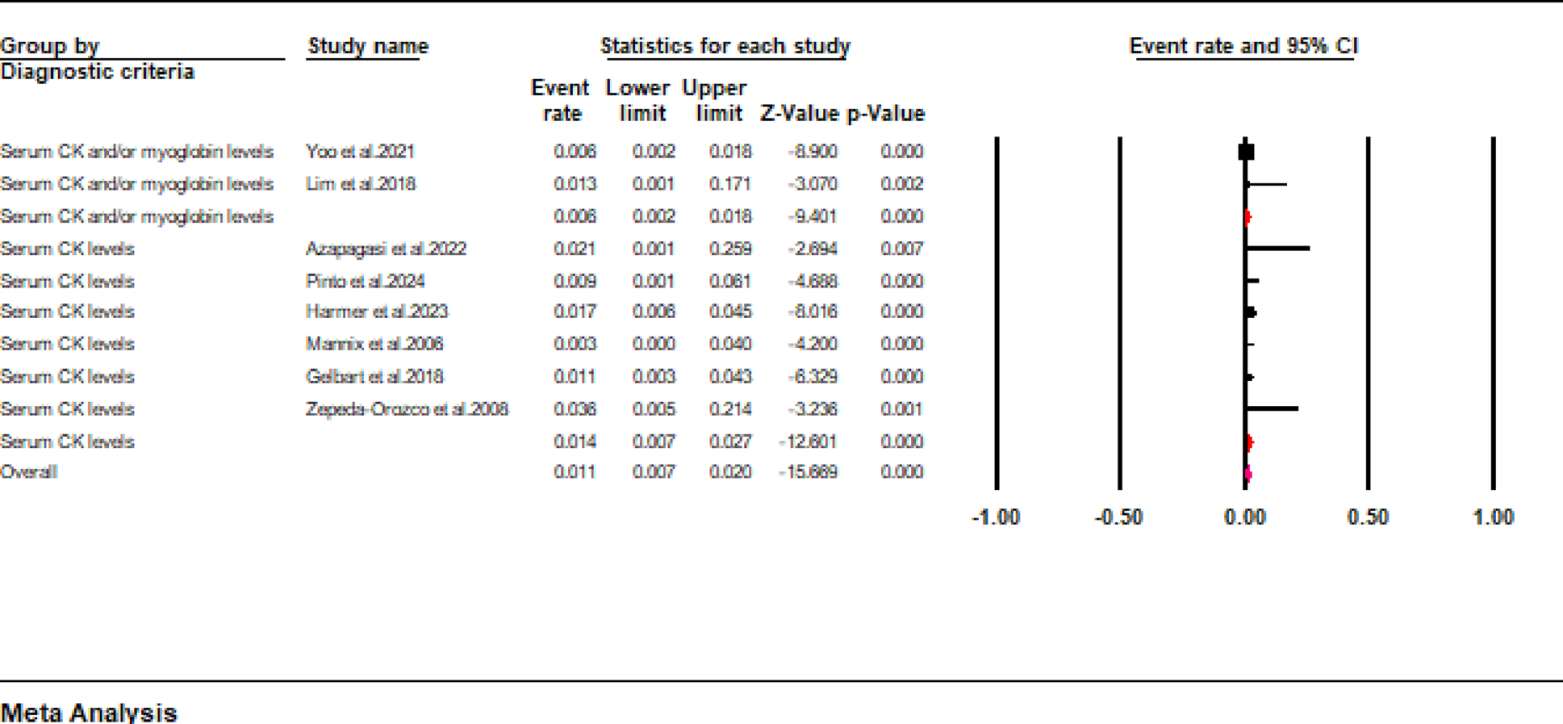
Forest plot showing the incidence of CKD stratified according to diagnostic criteria

On the other hand, our meta-analysis showed that the incidence of chronic kidney disease (CKD) in pediatric rhabdomyolysis was 1.1% (95% CI: 0.7 to 2; I^2^ = 0%) (Fig. 8). Furthermore, the subgroup analysis revealed that the incidences of CKD were 0.6% (95% CI: 0.1 to 17.1) and 1.4% (95% CI: 0.7 to 2.0) when rhabdomyolysis was diagnosed based on serum CK and/or myoglobin levels and serum CK levels, respectively (Fig. 9).

### Treatment of rhabdomyolysis

Table 1 shows the treatment strategies for pediatric patients with rhabdomyolysis. These treatments included hydration, urine alkalization with sodium bicarbonate or mannitol, dialysis, and kidney replacement therapy (KRT). From the qualitative review, we observed that KRT and hydration were the most commonly used treatment options for rhabdomyolysis in pediatric patients. However, due to the lack of sufficient data evaluating the effectiveness of these treatments, we were unable to conduct a meta-analysis.

### Kidney replacement therapy

According to Gelbart and colleagues, KRT was carried out in 16% of the patients (29/182) [20]. These authors reported that the most commonly used modality was the conventional veno-venous hemofiltration (CVVHF) (23/29), followed by the plasma filtration (3/29), high flux filter (2/29), and combination of CVVHF and plasma filtration (1/29). Patients that required KRT were found to longer mechanical ventilation (MV) hours (273 vs. 73; *p* < 0.001) and length of stay in the intensive care unit (ICU) than those who did not require KRT. On the other hand, Lim and colleagues found that out of 39 children with rhabdomyolysis, 14 developed AKI and 5 of them were treated using continuous kidney replacement therapy (CKRT) [24]. These five patients stayed in the hospital for a median of 7 days and they eventually recovered from AKI, with favorable long-term outcomes of rhabdomyolysis. In addition, Kuok and Chan (2021) reported that two patients who developed oliguria with significant fluid overload were treated with CKRT [18]. These patients were found to have normal serum creatinine, kidney sizes, and echogenic pattern after treatment. However, they developed hypertension during treatment and were treated using oral anti-hypertensive drugs.

### Dialysis

Park and colleagues reported that out of the six patients with AKI, 2 progressively worsened and had to be subjected to hemodialysis. These patients were reported to have had nephrotic syndrome for a long time and they were experiencing relapse during the rhabdomyolysis onset. Therefore, this finding suggests that hemodialysis might be necessary for rhabdomyolysis patients with underlying kidney diseases. Yoo et al. also found that 31.33% of 99 patients with AKI were treated with dialysis, with peritoneal dialysis being conducted in only one patient. In addition, Mannix and colleagues reported that one patient with rhabdomyolysis-induced AKI required dialysis. This patient survived and was reported to have normal creatinine levels at the final follow-up visit. Similarly, a study of 28 children with rhabdomyolysis reported that two of the 11 patients with AKI received hemodialysis and one received peritoneal dialysis. The investigators further stated that none of the patients died. Therefore, might be a necessary and effective treatment for rhabdomyolysis patients with deteriorating kidney function.

### Sensitivity analysis

A significant heterogeneity was noted in the pooled results showing the mortality rates of pediatric patients with rhabdomyolysis. Therefore, a sensitivity analysis was performed to investigate the sources of heterogeneity. After leaving out the results of Agharokh et al., Harmer et al., Kuok et al., Lim et al., Watanabe et al., Yoo et al., and Zepeda-Orozco et al., we noticed that the interstudy heterogeneity reduced from 94.7 to 9.8%. Similarly, be excluding the results of Agharokh et al., Chen et al., Gelbart et al., Kuok et al., Lim et al., Mannix et al., Park et al., Pinto et al., Watanabe et al., Watemberg et al., and Zepeda-Orozco et al., from the pooled results showing AKI incidence, the heterogeneity was lowered to 0% from 96%. These changes in heterogeneity imply that the heterogeneity was likely caused by variations in sample size, study design, diagnostic criteria for rhabdomyolysis, population sources, and patients age at baseline.

### Quality assessment outcomes

The results of quality assessment are shown in Table 2 (Appendix B, Supplementary Material 1). The overall quality assessment revealed that one study had a good methodological quality and the other fourteen had fair methodological quality. Most studies had fair methodological quality because they were conducted in single center, did not adjust confounders, and blinding of outcome assessors was not possible.

## Discussion

Rhabdomyolysis is a potentially fatal disorder resulting from skeletal muscle injury that compromises the integrity of the sarcolemma [8, 27]. In adults, trauma and drugs have been reported as some of the most common causes of rhabdomyolysis [6, 7]. However, the causes of rhabdomyolysis in children vary widely from those in adults. Therefore, the current meta-analysis investigated etiologies, treatment, and outcomes of pediatric rhabdomyolysis.

Our results have shown that infections are the most common cause of rhabdomyolysis in children, accounting for about 40.6% of rhabdomyolysis cases. This finding compares favorably with a previous review article, which reported that infections constitute about one-third of rhabdomyolysis cases observed in pediatric patients [8]. Various bacterial and viral infections have been associated with rhabdomyolysis; however, the available literature suggests that viral infections constitute the majority of infection-induced rhabdomyolysis. Indeed, Harmer and colleagues observed that infections were the predominant cause of rhabdomyolysis in pediatric patients in the UK, with 75% attributed to viral myositis [16]. Mannix et al. [19] also indicated that viral myositis constituted 38.2% of all rhabdomyolysis cases in juvenile patients. In addition, Park and colleagues found that out of 26 infection-related rhabdomyolysis cases, 65% were attributed to influenza, parainfluenza, coronavirus, adenovirus, rhinovirus, and enterovirus [13]. These findings are further supported by a previous state-of-the-art review, which reported that viral infections are one of the leading triggers for rhabdomyolysis in pediatric patients [28].

Several viral pathogens have been linked to the development of pediatric rhabdomyolysis, with the current study revealing that the influenza virus is the most common cause of infection-related rhabdomyolysis. The pathogenesis of rhabdomyolysis in patients with influenza virus is a matter of debate, but several hypotheses, including direct muscle invasion by the influenza virus, immunologic reaction “cytokine storm” resulting in collateral muscle damage, and circulating viral toxins causing direct muscle injury, have been proposed [29]. In addition to the influenza virus, parainfluenza, coronavirus, adenovirus, rhinovirus, enterovirus, and mycoplasma have been reported to cause rhabdomyolysis in children. The relationship between Mycoplasma and the development of rhabdomyolysis is not well-understood, but research shows that it results in potentially serious complications [30].

The pooled data also showed that exercise and trauma account for more than 10% of rhabdomyolysis in pediatric patients. However, most reports revealed that these etiologies tend to be more common in adolescents. For instance, Chen et al. [12] reported that trauma and exercise were the leading causes of rhabdomyolysis in children aged between 10 and 18 years. Yao et al. [14] also reported that strenuous exercise was the most common cause of rhabdomyolysis in the adolescent group (12 to 16 years). Despite these high incidences of exercise-induced rhabdomyolysis, there are several challenges in its diagnosis. One major challenge is that serum CK levels are naturally elevated after strenuous exercise in almost all normal people, potentially up to 10 times the normal level [31]. Another challenge is that increase in CK levels differ significantly among patients, and it is possible that a person may develop exertional rhabdomyolysis whereas another, expending the same energy under identical circumstances, does not [31]. Elevated temperature and humidity during physical activity could also lead to increased incidences of rhabdomyolysis [32].

Contrary to adult data [6, 7, 33], our study has shown that drugs are the cause of rhabdomyolysis in approximately 5.8% of pediatric patients. The substances that contribute to this drug-induced rhabdomyolysis vary from study to study. For instance, Park et al. [13] reported that two children developed drug-induced rhabdomyolysis as a result of using aripiprazole and diphenhydramine. The potential pathogenesis of rhabdomyolysis associated with aripiprazole involves the antagonistic action at serotonin 2 A (5HT2A) receptors in skeletal muscle [34]. However, there is no clear mechanism regarding the association of diphenhydramine and rhabdomyolysis. Other drugs reported to induce rhabdomyolysis in children include metformin, calcium channel blocker, selective serotonin reuptake inhibitor, weight loss pills taken together with salbutamol inhaler capsule, cocaine, d-Lisergic acid diethylamide, and 3,4methylenedioxymethamphetamine [16, 26].

AKI is a common and serious complication of rhabdomyolysis that can cause significant morbidity, mortality and progress to CKD if not managed early [35, 36]. In the present meta-analysis, we found that AKI developed in 21.3% of pediatric patients with rhabdomyolysis. However, the incidences of AKI seemed to vary depending on the patient selection criteria. Studies where patients were selected based on the myoglobin concentration alone reported AKI incidences of 13–50% [13, 21], while those that included patients based on elevated CK levels alone reported incidences between 7 and 49% [12, 14, 16, 18–20, 23, 25, 26]. We believe that this variation in the definition of rhabdomyolysis might have contributed to the significant heterogeneity observed in pooled outcomes. Nonetheless, there are three mechanisms that might explain the AKI observed in pediatric patients with rhabdomyolysis. First, myoglobin is usually released due to muscle injury. This myoglobin concentration increases around renal tubules, precipitating obstruction in the distal tubules. Second, fluid secreted in injured muscle tissues and the consequent stimulation of the renin-angiotensin-aldosterone pathway leads to renal vasoconstriction. Finally, the leakage of myoglobin might also promote the generation of reactive oxygen species and free radicals, contributing to immediate toxic action on the kidney tubular cells.

Since AKI is a life-threatening complication of rhabdomyolysis, it is vital to understand the predictors of AKI. However, only few studies in the current meta-analysis investigated the predictors of AKI. For instance, a logistic regression conducted by Yao and colleagues revealed that electrolyte disorder was a significant predictor of AKI but there was no association between age, gender, or peak CK levels and the development of AKI [14]. On the other hand, Pinto et al. [25] found that patients AKI were more likely to be older, have myoglobinuria, and have received intravenous bicarbonate, but there was no significant relationship between gender or peak CK levels and AKI. Collectively, these findings indicate that CK level and gender are not significant predictors of AKI in pediatric patients with rhabdomyolysis. However, further research in large-sample prospective trials is required to establish the independent predictors of AKI in pediatric rhabdomyolysis.

The present study also revealed that the incidence of CKD was low (1.1%), suggesting that CKD is a rare long-term kidney complication of pediatric rhabdomyolysis. Although factors predisposing rhabdomyolysis patients to CKD are not fully elucidated, it is believed that severe AKI, prolonged hypoperfusion, and presence of other complications might increase the risk. Indeed, an adult literature reported that at 3 months after rhabdomyolysis, the severity of AKI and high serum myoglobin levels were related with a decrease in estimated glomerular filtration rate, suggesting a potential kidney damage [37]. However, further research is needed to understand the predictive factors of CKD in pediatric rhabdomyolysis.

Currently, there is a lack of randomized clinical trials evaluating the best treatment in pediatric patients with rhabdomyolysis. However, regardless of the underlying etiology, treatments strategies for rhabdomyolysis include hydration, urine alkalization, dialysis, and kidney replacement therapy (KRT). Few studies in this meta-analysis have reported the efficacy of RRT in treating use children with rhabdomyolysis. For instance, a study of 39 Korean children reported that continuous RRT was effective in lowering CK and myoglobin levels, and patients subjected to this therapy showed complete recovery of kidney function [24]. This finding is collaborated by a meta-analysis of three RCTs which found that continuous RRT may provide benefits such as decrease in myoglobin levels, reduced duration of oliguria, and improved serum creatinine, blood urea nitrogen, and potassium levels compared to the conventional therapy [38]. The efficacy of hydration and urine alkalinization using sodium bicarbonate has also been reported in some of the included studies. Mannix et al. [19] found that higher fluid rates were associated with higher peak creatinine levels but no association was observed between bicarbonate therapy and peak creatinine level. Given the paucity of data, larger prospective studies are required to evaluate the effects of different treatment options on outcomes of children with rhabdomyolysis.

### Limitations

The current study has several limitations that are important to note during the interpretation of its findings. First, a high interstudy heterogeneity was recorded in most pooled outcomes. This heterogeneity might have been contributed by the variation in the definition of rhabdomyolysis, sample size, and geographical region. However, the effect of heterogeneity on the overall effect size was minimized using random-effects model. Second, all included studies were retrospective, indicating that our findings may have been influenced by bias inherent in these studies, since they may not have meticulously documented some patient information or may have overlooked certain patients owing to misclassification. Third, all the studies were conducted in single centers; therefore, results from these studies might have not been representative of the general pediatric population. Fourth, most studies had small sample sizes, meaning our results might be subject to small sample bias. Fifth, only articles written in English were included in the analysis. Therefore, it is possible that our study was subject to selection bias as data from non-English literature was intentionally omitted. Sixth, this meta-analysis exclusively evaluated pediatric rhabdomyolysis; however, we acknowledge that the lack of direct comparison with adult populations might have limited the broader generalizability of our study. Moreover, including separate data for adult populations was beyond the scope of this review and would have confounded our analysis and introduced significant clinical heterogeneity. Therefore, studies directly comparing adult and pediatric rhabdomyolysis are needed to better inform age-specific differences in etiologies, treatment, and outcomes. Finally, due to paucity of data, we did not carry out any meta-analysis evaluating the efficacy of different treatment strategies or predictive factors for AKI. As such, future studies reporting these findings can help to address this limitation.

## Conclusion

In summary, our research indicated that infection was the predominant cause of rhabdomyolysis in children, followed by trauma and exercise. Other etiologies such as drugs, seizures, connective tissue disorders, muscular dystrophy, metabolic anomalies, burns, sepsis, and multiorgan failure comprise less than 10% of rhabdomyolysis cases but should not be overlooked as potential etiologies of rhabdomyolysis in children. Moreover, we found that AKI occurs in about 21.3% of children with rhabdomyolysis, but the mortality rate and CKD incidence are low. These findings imply that although AKI is a common complication, the prognosis of pediatric patients with rhabdomyolysis is good and very few patients progress to CKD. We also found limited data on the treatment strategies for children with rhabdomyolysis. Therefore, large-sample prospective trials are needed to identify effective treatments for pediatric rhabdomyolysis.

## Supplementary Information

Below is the link to the electronic supplementary material.

Supplementary Material 1

Supplementary Material 2

## Data Availability

The data that support the findings of this study are available from the corresponding author upon reasonable request.

## Abbreviations

AKI: Acute Kidney Injury
CK: Creatine Kinase
CKD: Chronic Kidney Disease
CMA: Comprehensive Meta-Analysis
KRT: Kidney Replacement Therapy
NOS: Newcastle-Ottawa Scale
PECO: Population, Exposure, Comparison, Outcome
PICO: Patient, Intervention, Comparison, Outcome
PRISMA: Preferred Reporting Items for Systematic Reviews and Meta-Analyses
PROSPERO: International Prospective Register of Systematic Reviews
RCT: Randomized Controlled Trial
RRT: Renal Replacement Therapy
