# Supplementary material for: Pediatric rhabdomyolysis: a systematic review and meta-analysis of etiologies, management, and outcomes
Source: BMC Pediatr. 2025 Oct 27;25:866. doi: 10.1186/s12887-025-06081-x (PMC12560446; doi:10.1186/s12887-025-06081-x)
Supplement: Supplementary file 1 — Supplementary Material 1 [file 12887_2025_6081_MOESM1_ESM.docx]

**Appendix A: Search Strategy**

**PubMed**

**[All Fields]** (etiology OR cause OR etiologies OR causes OR aetiology OR primary cause OR primary diagnosis) AND (rhabdomyolysis OR myoglobinuria OR muscle breakdown OR rhabdo OR acute muscle breakdown syndrome) AND (children OR infants OR pediatric OR young people) AND (acute kidney injury OR acute renal failure OR chronic kidney disease OR chronic renal failure OR mortality OR death OR fatality)

**[Title/Abstract]** (etiology OR cause OR etiologies OR causes OR aetiology OR primary cause OR primary diagnosis) AND (rhabdomyolysis OR myoglobinuria OR muscle breakdown OR rhabdo OR acute muscle breakdown syndrome) AND (children OR infants OR pediatric OR young people) AND (acute kidney injury OR acute renal failure OR chronic kidney disease OR chronic renal failure OR mortality OR death OR fatality)

**Cochrane Library**

(etiology OR cause OR etiologies OR causes OR aetiology OR primary cause OR primary diagnosis) AND (rhabdomyolysis OR myoglobinuria OR muscle breakdown OR rhabdo OR acute muscle breakdown syndrome) AND (children OR infants OR pediatric OR young people) AND (acute kidney injury OR acute renal failure OR chronic kidney disease OR chronic renal failure OR mortality OR death OR fatality)

**EMBASE**

(etiology OR cause OR etiologies OR causes OR aetiology OR primary cause OR primary diagnosis) AND (rhabdomyolysis OR myoglobinuria OR muscle breakdown OR rhabdo OR acute muscle breakdown syndrome) AND (children OR infants OR pediatric OR young people) AND (acute kidney injury OR acute renal failure OR chronic kidney disease OR chronic renal failure OR mortality OR death OR fatality)

**Web of Science**

(etiology OR cause OR etiologies OR causes OR aetiology OR primary cause OR primary diagnosis) AND (rhabdomyolysis OR myoglobinuria OR muscle breakdown OR rhabdo OR acute muscle breakdown syndrome) AND (children OR infants OR pediatric OR young people) AND (acute kidney injury OR acute renal failure OR chronic kidney disease OR chronic renal failure OR mortality OR death OR fatality)

**Google Scholar**

(etiology OR cause OR etiologies OR causes OR aetiology OR primary cause OR primary diagnosis) AND (rhabdomyolysis OR myoglobinuria OR muscle breakdown OR rhabdo OR acute muscle breakdown syndrome) AND (children OR infants OR pediatric OR young people) AND (acute kidney injury OR acute renal failure OR chronic kidney disease OR chronic renal failure OR mortality OR death OR fatality)

**Appendix B: Quality Appraisal**

**Table 2: Methodological Quality According Newcastle Ottawa Scale**

| Study ID | Exposed cohort representativeness | Non-exposed cohort selection | Exposure verification | Initial outcome absence | Cohort comparability (Design/Analysis adjusted for confounders) | Outcome evaluation | Sufficient follow-up | Cohort follow-up | Total score | Overall quality |
| --- | --- | --- | --- | --- | --- | --- | --- | --- | --- | --- |
| Chen et al.2013(12) | 0 | 1 | 1 | 1 | 0 | 0 | 1 | 1 | 5 | Fair |
| Park et al.2018(13) | 0 | 1 | 1 | 1 | 0 | 0 | 1 | 1 | 5 | Fair |
| Yao et al.2020(14) | 0 | 1 | 1 | 1 | 0 | 0 | 1 | 1 | 5 | Fair |
| Agharokh et al.2022(15) | 0 | 1 | 1 | 1 | 2 | 0 | 1 | 1 | 7 | Good |
| Harmer et al.2023(16) | 0 | 1 | 1 | 1 | 0 | 0 | 1 | 1 | 5 | Fair |
| Yoo et al.2021(17) | 0 | 1 | 1 | 1 | 0 | 0 | 1 | 1 | 5 | Fair |
| Kuok et al.2021(18) | 0 | 1 | 1 | 1 | 0 | 0 | 1 | 1 | 5 | Fair |
| Mannix et al.2006(19) | 0 | 0 | 1 | 1 | 2 | 0 | 1 | 1 | 6 | Fair |
| Gelbart et al.2018(20) | 0 | 1 | 1 | 1 | 0 | 0 | 1 | 1 | 5 | Fair |
| Watanabe, 2001(21) | 0 | 1 | 1 | 1 | 0 | 0 | 1 | 1 | 5 | Fair |
| Watemberg et al.2000(22) | 0 | 0 | 1 | 1 | 0 | 0 | 1 | 1 | 4 | Fair |
| Zepeda-Orozco et al.2008(23) | 0 | 1 | 1 | 1 | 0 | 0 | 1 | 1 | 5 | Fair |
| Lim et al.2018(24) | 0 | 1 | 1 | 1 | 0 | 0 | 1 | 1 | 5 | Fair |
| Pinto et al.2024(25) | 0 | 1 | 1 | 1 | 0 | 0 | 1 | 1 | 5 | Fair |
| Azapağası et al.2022(26) | 0 | 1 | 1 | 1 | 0 | 0 | 1 | 1 | 5 | Fair |
